# Complete Genome Sequence of a *Lily virus X* Isolate from Japan

**DOI:** 10.1128/genomeA.01462-17

**Published:** 2018-01-25

**Authors:** Takamichi Nijo, Yukari Okano, Masayoshi Kondo, Hiroaki Okuhara, Hiroyo Sekimura, Yuji Fujimoto, Naoi Hosoe, Kensaku Maejima, Yasuyuki Yamaji, Shigetou Namba

**Affiliations:** a Department of Agricultural and Environmental Biology, Graduate School of Agricultural and Life Sciences, The University of Tokyo, Tokyo, Japan; b Biotechnology Division, Niigata Agricultural Research Institute, Niigata, Japan

## Abstract

The complete genome sequence of *Lily virus X* (LVX), which infects lilies, was determined for the first time from lilies in Japan. As with previous reports, the genome of the Japanese LVX isolate lacked an AUG start codon for the triple gene block protein 3-like region.

## GENOME ANNOUNCEMENT

*Lily virus X* (LVX) is a member of the genus *Potexvirus* in the family *Alphaflexiviridae* (1, 2). LVX possesses flexuous filamentous particles and a single-stranded positive-sense RNA genome. LVX was first reported in a symptomless lily (*Lilium formosanum*) from England (3) and is known to occur in the Netherlands and the United States (4, 5). To date, the complete genome of one LVX isolate from the Netherlands has been reported (6). Although the reported genome organization of LVX was similar to that of other members of the genus *Potexvirus*, it lacked an AUG start codon for the triple gene block protein 3 (TGBp3), one of the movement proteins (4, 6). In Japan, although LVX is known to be the causative agent of lily necrosis when associated with lily mottle virus (7) and has also been detected from symptomless lily cultivars (8), sequence information on Japanese LVX isolates has not been reported. Here, we report for the first time a complete genome sequence of LVX isolated from a lily in Japan.

Lily plants (*Lilium* cv. Acapulco) were collected in the Chubu District of Japan in 2016. Total RNA was extracted from leaves using Isogen reagent (Nippon Gene, Japan). A paired-end sequencing cDNA library was constructed from the extracted RNA using a TruSeq RNA sample prep kit v.2 (Illumina, USA) and sequenced using a MiSeq instrument (Illumina) and a MiSeq reagent kit v.2 (500 cycles). After quality control and trimming using Trimmomatic v.0.36 software (9), the reads were *de novo* assembled using Trinity v.2.3.2 software (10). The assembled contigs were subjected to a BLASTn search (11) against the GenBank database, and a contig showing sequence identity with LVX (GenBank accession number AJ633822) was obtained. To determine the complete genome sequence, the 5′ and 3′ ends of the genome were amplified by rapid amplification of cDNA ends (RACE) using a Gene Racer kit (Invitrogen, USA). The amplified fragments were cloned into pCR-Blunt II-TOPO vector (Invitrogen) and sequenced.

The complete genome sequence of the Japanese isolate of LVX (LVX-J) was 5,824 nucleotides (nt) long, excluding the poly(A) tail at its 3′ end. It contained four open reading frames (ORFs) encoding RNA-dependent RNA polymerase (RdRp) (nt 73 to 3969), TGBp1 (nt 3999 to 4649), TGBp2 (nt 4649 to 4975), and coat protein (CP) (nt 5110 to 5715). The TGBp3-like region (nt 4893 to 5129) lacked an AUG start codon, as in the case of the previously reported isolate (6). The respective percent identities of RdRp, TGBp1, TGBp2, TGBp3, and CP with those of the reported LVX isolate (GenBank accession number AJ633822) were 99.4%, 98.8%, 99.7%, 99.6%, and 99.3% at the nucleotide level and 99.3%, 98.6%, 99.1%, 97.4%, and 99.0% at the amino acid level, respectively. These results indicate that LVX-J is closely related to the reported LVX isolate from the Netherlands. Because lilies are propagated vegetatively from bulbs, a genetically identical strain of LVX might be spread worldwide by LVX-infected lily bulbs.

### Accession number(s).

The complete genome sequence of *Lily virus X* isolate J has been deposited in the DNA Data Bank of Japan and GenBank under the GenBank accession number LC335818.
